# Supplementary material for: Cell-based and isoform-selective G protein-coupled receptor kinase assays for comprehensive inhibitor evaluation
Source: Commun Biol. 2026 Jan 16;9:287. doi: 10.1038/s42003-026-09568-0 (PMC12920735; doi:10.1038/s42003-026-09568-0)
Supplement: Supplementary file 2 — Supplementary Information [file 42003_2026_9568_MOESM2_ESM.pdf]

Supplementary Information

Cell-based and isoform-selective G protein-coupled receptor kinase assays for comprehensive inhibitor evaluation

Nina K. Blum, Manuela C. Kiefer, Angelika Decker, Laura Klement, Edda S. F. Matthees, Verena Weitzel, Falko Nagel, Babu Joseph, Julia Drube, David Uehling, Carsten Hoffmann, Stefan Schulz

Supplementary material including Supplementary Tables 1-4 and Supplementary Figures 1-4 as well as respective legends

Supplementary Table 1. Influence of different GRK isoforms on agonist-induced receptor phosphorylation of T360/S364 as determined by 7TM phosphorylation assays

Cell line	$logEC_{50}$	E_{max} (% of ISO)
Control-β2	-6.00 ± 0.04	104 ± 7.6
ΔGRK5/6-β2	-6.00 ± 0.05	73.4 ± 4.1
ΔGRK2/3-β2	-6.04 ± 0.08	66.1 ± 8.2
ΔQ-GRK-β2	N/A	3.4 ± 1.8
**Cell line**	**$logEC_{50}$**	**E_{max} (% of ISO)**
Control-β2	-5.85 ± 0.04	100 ± 5.7
ΔQ+GRK2-β2	-5.58 ± 0.17	60.2 ± 2.3
ΔQ+GRK3-β2	-5.75 ± 0.06	82.7 ± 5.1
ΔQ+GRK5-β2	-5.96 ± 0.05	81.7 ± 4.2
ΔQ+GRK6-β2	-5.77 ± 0.05	88.8 ± 3.6
ΔQ-GRK-β2	N/A	-0.1 ± 0.3

HEK293 cells were treated with increasing concentrations of isoproterenol (ISO) (30 min, 37°C). Data represent means ± SEM of at least n=4 independent experiments.

Supplementary Table 2. GRK inhibition of ISO-induced β2 phosphorylation of T360/S364 in ΔGRK2/3 and ΔGRK5/6 cells as determined by 7TM phosphorylation assays

Inhibitor	ΔGRK5/6-β2		ΔGRK2/3-β2	
	$\log IC_{50}$	maximal inhibition of ISO-induced phosphorylation	$\log IC_{50}$	maximal inhibition of ISO-induced phosphorylation
CCG215022	>(-4.5)	12%	>(-4.5)	8%
CCG258208	-5.11 ± 0.08	100%	>(-4.5)	33%
CCG258747	-5.06 ± 0.07	100%	>(-4.5)	35%
CCG273441	>(-4.5)	0%	-7.57 ± 0.07	100%
Compound 8h	-6.16 ± 0.05	99%	>(-4.5)	0%
Compound 10a	-4.73 ± 0.09	73%	-5.46 ± 0.16	99%
Compound 18	>(-4.5)	18%	-6.12 ± 0.12	97%
Compound 19	>(-4.5)	10%	-6.54 ± 0.06	100%
Compound 101	-5.01 ± 0.18	93%	>(-4.5)	28%
Compound 707	>(-4.5)	5%	>(-4.5)	17%
GSK180736	>(-4.5)	0%	>(-4.5)	21%
KR-39038	>(-4.5)	0%	>(-4.5)	12%
LDC9728	-5.80 ± 0.07	97%	-6.33 ± 0.08	100%

HEK293 cells were preincubated with increasing concentrations of GRK inhibitor (30 min, 37°C) prior to agonist stimulation (10 µM ISO, 30 min, 37°C). Maximal inhibition of ISO-induced phosphorylation was determined at the highest concentration tested (30 µM). Data represent means ± SEM of n=5 independent experiments.

Supplementary Table 3. List of $\log IC_{50}$ values of inhibitors tested in ΔQ-GRK cells stably expressing β2 and overexpressing one GRK isoform as determined by plate 7TM phosphorylation assays

Inhibitor	ΔQ+GRK2-β2		ΔQ+GRK3-β2	
	**$\log IC_{50}$**	**maximal inhibition of ISO-induced phosphorylation**	**$\log IC_{50}$**	**maximal inhibition of ISO-induced phosphorylation**
CCG258747	-5.09 ± 0.04	94%	-5.38 ± 0.20	96%
CCG273441	>(-4.5)	0%	>(-4.5)	31%
Compound 8h	-5.68 ± 0.19	100%	-5.49 ± 0.11	99%
Compound 18	>(-4.5)	11%	>(-4.5)	1%
Compound 19	-4.72 ± 0.12	61%	>(-4.5)	44%
Compound 101	-4.65 ± 0.16	69%	-5.36 ± 0.18	100%
LDC9728	-5.61 ± 0.08	98%	-5.88 ± 0.06	97%
**Inhibitor**	**ΔQ+GRK5-β2**		**ΔQ+GRK6-β2**	
	**$\log IC_{50}$**	**maximal inhibition of ISO-induced phosphorylation**	**$\log IC_{50}$**	**maximal inhibition of ISO-induced phosphorylation**
CCG258747	>(-4.5)	50%	>(-4.5)	56%
CCG273441	-7.31 ± 0.15	100%	-7.25 ± 0.10	100%
Compound 8h	>(-4.5)	0%	>(-4.5)	0%
Compound 18	-4.95 ± 0.19	77%	-5.07 ± 0.18	97%
Compound 19	-5.83 ± 0.10	98%	-5.96 ± 0.11	100%
Compound 101	>(-4.5)	24%	>(-4.5)	0%
LDC9728	-6.53 ± 0.10	100%	-5.73 ± 0.19	100%

HEK293 cells were preincubated with increasing concentrations of GRK inhibitor (30 min, 37°C) prior to agonist stimulation (10 μM ISO, 30 min, 37°C). Maximal inhibition of ISO-induced phosphorylation was determined at the highest concentration tested (30 μM). Data represent means ± SEM of n=5 independent experiments.

Supplementary Table 4. Impact of GRK inhibitors on receptor phosphorylation and internalization as determined by Western blot and immunocytochemistry stainings

**Receptor**	**Condition**	**Phosphorylation** % of agonist control	**Internalization** % of agonist control
β2	vehicle control	3.8 ± 1.2	10.7 ± 3.2
	agonist control	100 ± 8.0	100 ± 0.9
	Compound 8h	82.8 ± 6.5	95.3 ± 3.8
	Compound 18	74.7 ± 4.3	93.7 ± 6.1
	Compound 8h + Compound 18	0.9 ± 0.3	13.8 ± 3.3
MOP	vehicle control	2.2 ± 0.6	13.2 ± 1.5
	agonist control	100 ± 7.6	100 ± 1.2
	Compound 8h	35.5 ± 3.0	29.7 ± 1.3
	Compound 18	44.3 ± 7.6	97.4 ± 2.9
	Compound 8h + Compound 18	1.5 ± 0.6	26.9 ± 1.9
S1P1	vehicle control	3.0 ± 1.3	6.7 ± 0.4
	agonist control	100 ± 6.5	100 ± 5.3
	Compound 8h	58.0 ± 7.6	15.7 ± 2.1
	Compound 18	85.3 ± 4.9	90.3 ± 4.8
	Compound 8h + Compound 18	6.1 ± 2.5	8.0 ± 1.7
V2	vehicle control	3.7 ± 1.5	13.2 ± 0.7
	agonist control	100 ± 6.6	100 ± 3.4
	Compound 8h	46.3 ± 3.7	39.3 ± 2.2
	Compound 18	85.7 ± 6.7	91.6 ± 5.9
	Compound 8h + Compound 18	2.8 ± 0.6	13.4 ± 5.1

HEK293 cells were treated with either compound 8h, compound 18 or combinations thereof (30 µM, 30 min, 37°C) prior to agonist stimulation (30 min, 37°C). Data represent means ± SEM of quantified signals from n=5 independent experiments.

Supplementary Figure 1: Linearity of the 7TM phosphorylation assay.

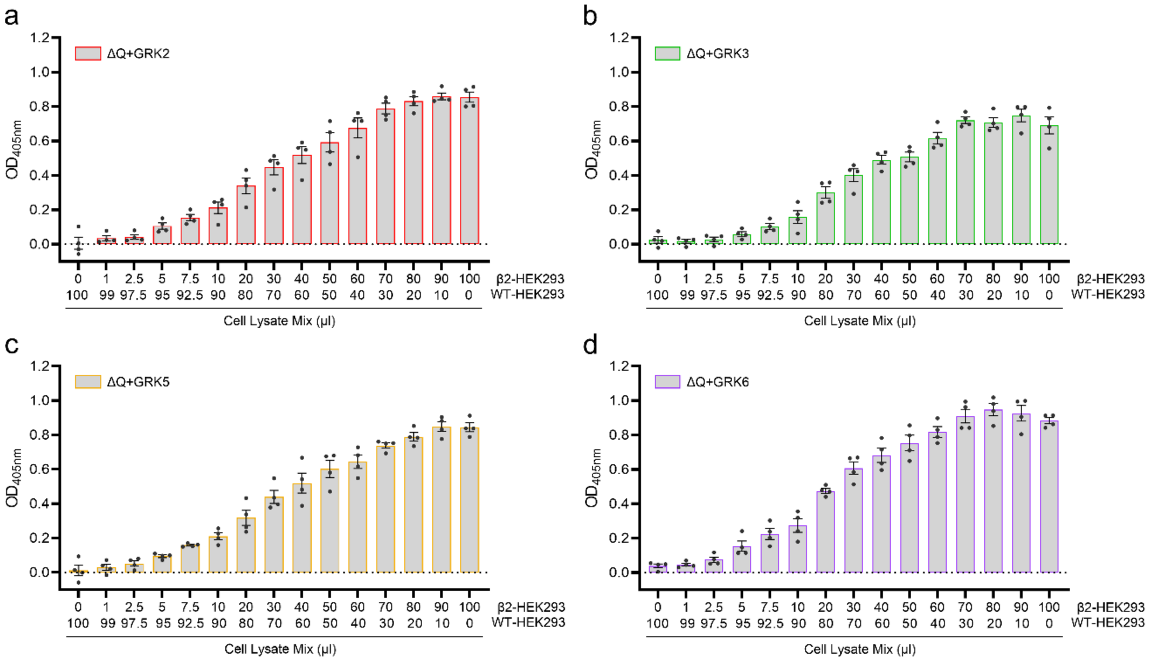

HEK293 ΔQ-GRK cell lines stably expressing the β2 and one GRK isoform or wildtype (WT) HEK293 cells were stimulated with 10 µM isoproterenol (30 min, 37°C) and lysed with detergent buffer. Different dilutions ranging from 0 µl to 100 µl were generated by mixing lysates from β2-expressing and WT cells. Receptor phosphorylation at the T360/S364 site was assessed using the standard 7TM phosphorylation assay protocol in ΔQ+GRK2 (**a**), ΔQ+GRK3 (**b**), ΔQ+GRK5 (**c**) and ΔQ+GRK6 (**d**). Raw optical density (OD) values were corrected for background and bar graphs display mean ± SEM from n=4 independent experiments performed in duplicates.

Supplementary Figure 2: Raw optical density (OD) data obtained from HEK293 Control and ΔQ-GRK cell lines overexpressing one GRK isoform.

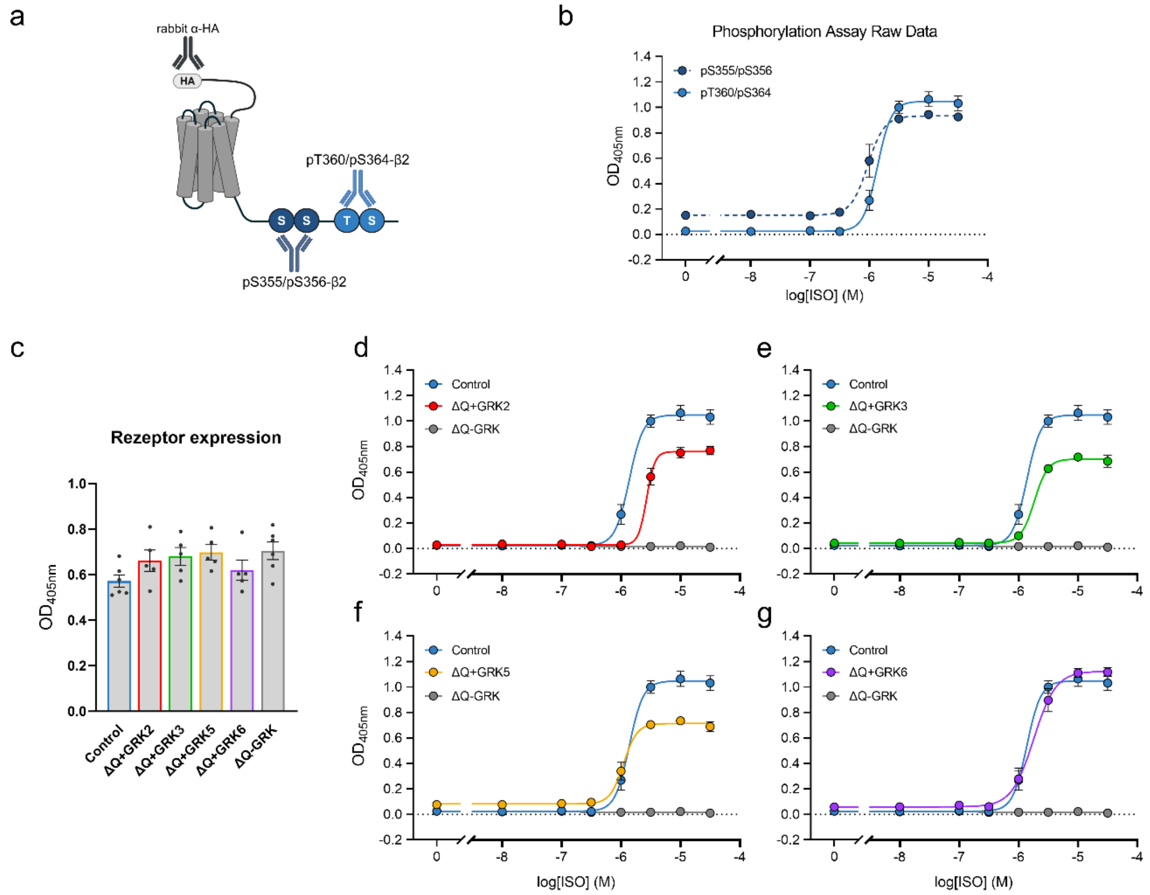

(**a**) Schematic representation of the β2 adrenergic receptor (β2) indicating the antibody binding sites targeting two intracellular phosphorylation sites (Created in BioRender. Blum, N. (2026) https://BioRender.com/yyb2lki). (**b**) HEK293 cells stably expressing β2 were stimulated with increasing concentrations of isoproterenol (ISO) (30 min, 37°C). Phosphorylation of the S355/S356 and T360/S364 site were assessed using the 7TM phosphorylation assay. (**c-f**) Graphs show the raw OD values corresponding to the data presented in Figure 4. Concentration-response curves from HEK293 Control and ΔQ-GRK cells are compared with ΔQ+GRK2 (**c**), ΔQ+GRK3 (**d**), ΔQ+GRK5 (**e**) and ΔQ+GRK6 (**f**). (**g**) Bar graph depicts the mean raw OD values from each experiment obtained using the anti-HA antibody, therefore representing the receptor expression in the respective cell lines. All graphs display the mean ± SEM of raw OD values corrected for background and before normalization of at least n=5 independent experiments performed in duplicates.

Supplementary Figure 3: Immunostaining after treatment with the covalent GRK5 inhibitor CCG273441.

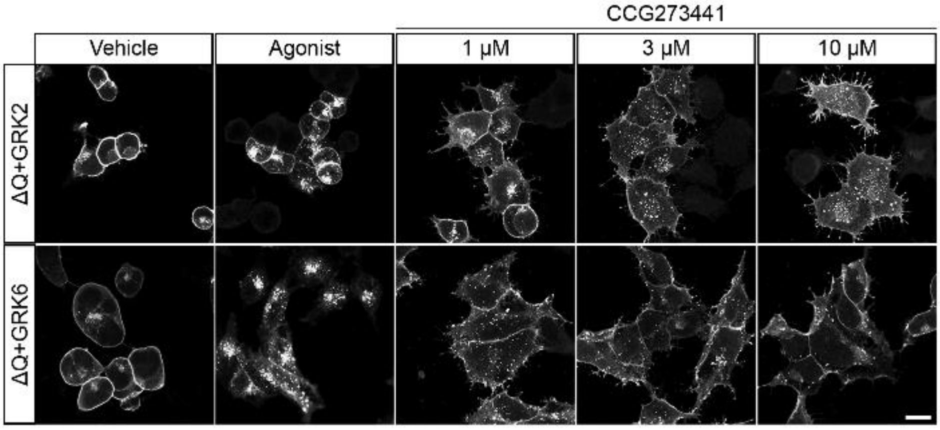

HEK293 cells co-expressing the vasopressin receptor 2 (V2) and GRK2 or GRK6 on a quadruple knockout background (ΔQ+GRK2 or ΔQ+GRK6) were treated with 1 µM vasopressin (Agonist) or were left untreated (Vehicle) (30 min, 37°C). To inhibit the GRK isoforms 5 and 6, cells were preincubated with increasing concentrations of CCG273441 (30 min, 37°C) prior to agonist stimulation. Hemagglutinin (HA)-tagged receptors were stained using anti-HA antibody. Immunofluorescent images represent one of n=2 independent experiments. The scale bar corresponds to 20 µm.

Supplementary Figure 4: Ligand-induced β-arrestin2 recruitment to the muscarinic acetylcholine receptor 5 (M5) or parathyroid hormone 1 receptor (PTH1) after the addition of GRK inhibitors.

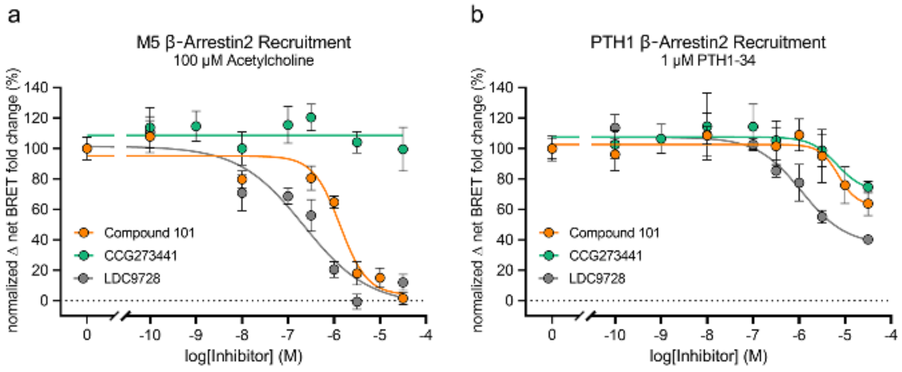

(**a**, **b**) HEK293 cell lines expressing NanoLuciferase-tagged M5 (**a**) or PTH1 (**b**) and Halo-tagged β-arrestin2 were treated with increasing concentrations of Compound 101, CCG273441 or LDC9728 (30 min, 37°C) and stimulated with their respective agonist (**a**: 100 μM acetylcholine, **b**: 1 μM PTH1-34) (30 min, 37°C). Data of the NanoBRET β-arrestin recruitment assay are depicted as Δ net BRET fold change normalized to the mean value of the condition without GRK inhibitor. Graphs represent mean ± SEM of n=4 independent experiments performed in triplicates.

Supplementary Figure 5: Uncropped Western blot images corresponding to the results shown in Figure 4b.

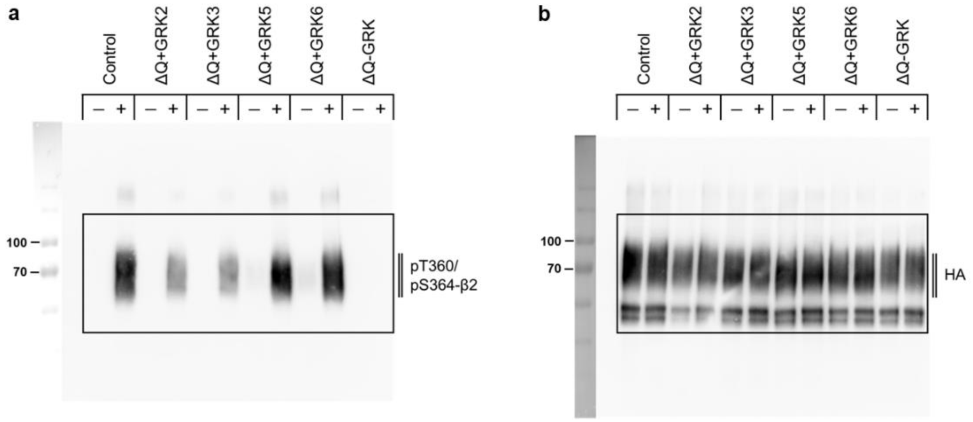

Cell lines were stimulated with 10 µM isoproterenol (+) or were left untreated (-). Blots were probed with a phosphosite-specific antibody against the β2 adrenergic receptor (pT360/pS364-β2) (**a**) or a phosphorylation-independent antibody against the hemagglutinin-tag (HA) (**b**). Images illustrate one representative of n=5 replicates with molecular weight markers. A frame highlights the region shown in the main manuscript figure.

Supplementary Figure 6: Uncropped Western blot images corresponding to the results shown in Figure 7.

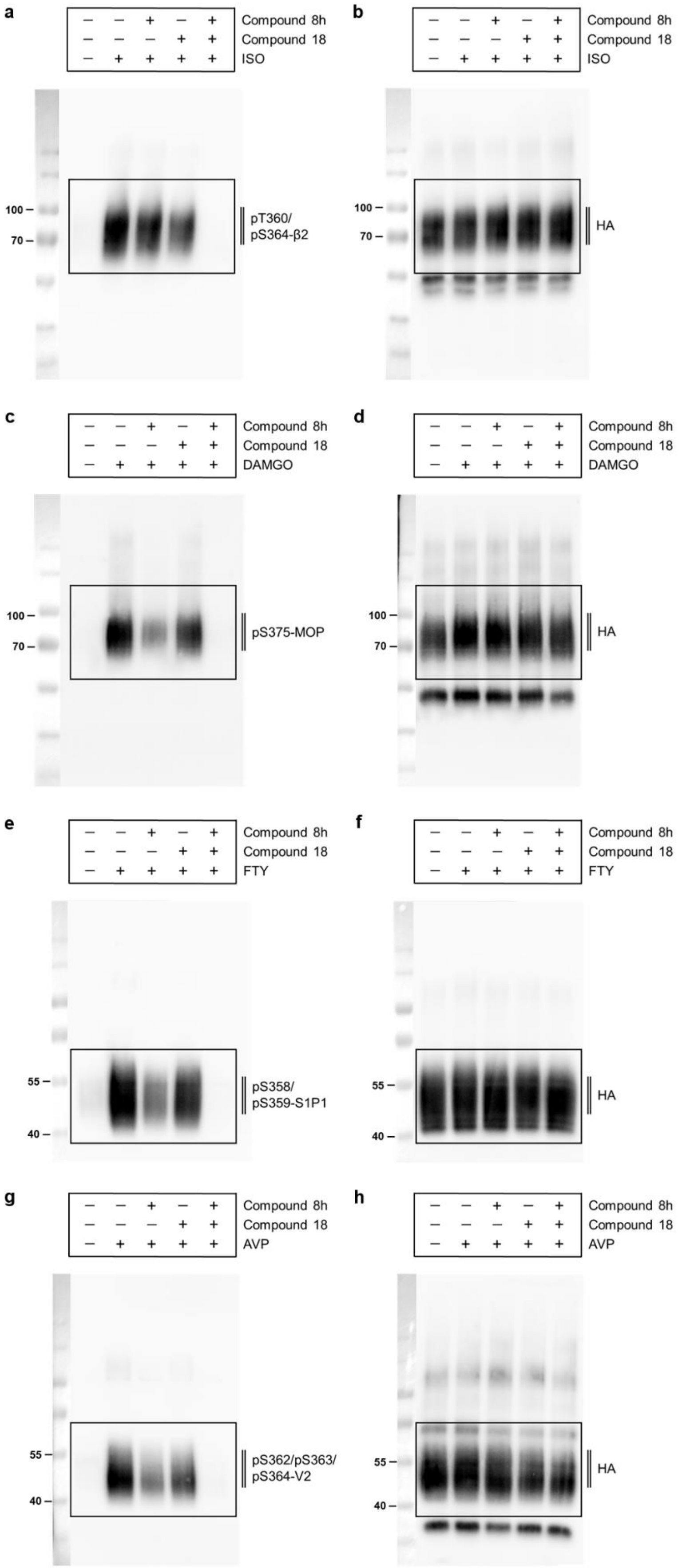

Cell lines stably expressing a GPCR of interest were treated with the indicated compounds and agonists. Blots were probed with phosphosite-specific antibodies against the β2 adrenergic receptor (pT360/pS364-β2) (**a**), the μ-opioid receptor (pS375-MOP) (**c**), the sphingosine-1-phosphate receptor 1 (pS358/S359-S1P1) (**e**) or the vasopressin receptor 2 (pS362/pS363/pS364-V2) (**g**). Receptor expression was assessed using anti-HA antibodies (HA) (**b, d, f, h**). Images illustrate one representative of n=5 replicates with molecular weight markers. A frame highlights the region shown in the main manuscript figure.
