# Supplementary material for: Cell-based and isoform-selective G protein-coupled receptor kinase assays for comprehensive inhibitor evaluation
Source: Commun Biol. 2026 Jan 16;9:287. doi: 10.1038/s42003-026-09568-0 (PMC12920735; doi:10.1038/s42003-026-09568-0)
Supplement: Supplementary file 3 — Description of Additional Supplementary Files [file 42003_2026_9568_MOESM3_ESM.pdf]

Description of Additional Supplementary File

File name: Supplementary Data

Description: The source data for all graphs depicted in the paper.
